# Cholesterol Oxidase Binds TLR2 and Modulates Functional Responses of Human Macrophages

**DOI:** 10.1155/2014/498395

**Published:** 2014-07-08

**Authors:** Katarzyna Bednarska, Michal Kielbik, Zofia Sulowska, Jaroslaw Dziadek, Magdalena Klink

**Affiliations:** Institute of Medical Biology, Polish Academy of Sciences, Lodowa 106, 93-232 Lodz, Poland

## Abstract

Cholesterol oxidase (ChoD) is considered to be an important virulence factor for *Mycobacterium tuberculosis* (Mtb), but its influence on macrophage activity is unknown. Here we used *Nocardia erythropolis* ChoD, which is very similar to the Mtb enzyme (70% identity at the amino-acid level), to evaluate the impact of bacterial ChoD on the activity of THP-1-derived macrophages in vitro. We found that ChoD decreased the surface expression of Toll-like receptor type 2 (TLR2) and complement receptor 3 (CR3) on these macrophages. Flow cytometry and confocal microscopy showed that ChoD competed with lipoteichoic acid for ligand binding sites on TLR2 but not on CR3, suggesting that ChoD signaling is mediated via TLR2. Binding of ChoD to the membrane of macrophages had diverse effects on the activity of macrophages, activating p38 mitogen activated kinase and stimulating production of a large amount of interleukin-10. Moreover, ChoD primed macrophages to enhance the production of reactive oxygen species in response to the phorbol myristate acetate, which was reduced by “switching off” TLR-derived signaling through interleukin-1 receptor-associated kinases 1 and 4 inhibition. Our study revealed that ChoD interacts directly with macrophages via TLR2 and influences the biological activity of macrophages during the development of the initial response to infection.

## 1. Introduction

Cholesterol oxidase (ChoD) is a flavoenzyme found in some bacteria species, including those of the genera* Mycobacterium*,* Rhodococcus*,* Nocardia*,* Arthrobacter, Pseudomonas, Corynebacterium, *and* Streptomyces.* This enzyme can be produced by bacteria in three forms: intracellular, extracellular, and membrane bound. Many bacteria produce ChoD as both an extracellular enzyme and a cell surface membrane-associated form [1–4]. It has been postulated that nonpathogenic bacteria use ChoD to degrade cholesterol, whereas pathogenic bacteria utilize it to infect the host macrophages, reflecting its ability to alter the physical structure of the lipid membrane [5].

It is well accepted that in both fast- and slow-growing mycobacteria, the initial step of cholesterol degradation—the conversion of cholesterol to its 3-keto-4-ene derivative, cholestenone—is based on hydroxysteroid dehydrogenase rather than ChoD [6–8]. Instead, ChoD in* Mycobacterium tuberculosis* (Mtb) appears to be an important virulence factor, since growth and survival of Mtb mutants defective in the synthesis of this enzyme (Δ*choD* Mtb) are attenuated in THP-1-derived macrophages, mice peritoneal macrophages, and lungs and spleens of infected mice [9, 10]. We have recently shown that the production of toxic radicals, nitric oxide (NO), and reactive oxygen species (ROS) by THP-1-derived macrophages infected with the Δ*choD* Mtb mutant is significantly lower compared to those infected with the wild-type strain. Moreover, an intact* choD* gene is required for Mtb to suppress activation of Toll-like (TLR2) and complement receptor 3 (CR3) signaling pathways [10].

Nguyen and Taub [11] demonstrated that direct treatment of monocytes with ChoD from* Pseudomonas fluorescens* affects the conformation and function of chemokine receptors by depleting cholesterol from the cell membrane. This emphasizes the role of cholesterol-rich lipid domains of the cell membrane in preserving the appropriate structure and activity of receptors, including TLRs and chemokine receptors [12]. Moreover, cholesterol-rich lipid domains are important components of membrane-bound enzymes responsible for ROS production in neutrophils [13]. However, the mechanism by which bacterial ChoD directly interacts with macrophages has been poorly understood and requires clarification.

We hypothesized that ChoD may play an important role in modulating macrophage biological activity and, thus, may represent an important component of bacterial virulence. Therefore, understanding the pathways and mechanisms involved in macrophage reactivity upon contact with extracellular ChoD may be important with respect to the possibility of regulating the host immune system response after infection with bacteria.

Here we used commercially available* Nocardia erythropolis* ChoD, which is highly similar to its ortholog [14] in Mtb, to study the impact of bacterial ChoD on the biological activity of THP-1-derived macrophages. We investigated the direct interactions between ChoD and human macrophages, the influence of ChoD on the expression of the pattern-recognition receptors, TLR2 and CR3, and the phosphorylation of key signaling kinases involved in cell activation.

## 2. Materials and Methods

### 2.1. Chemicals and Antibodies

Trypsin/EDTA (1x, 0.05% solution), RPMI-1640 medium containing 1 mM sodium pyruvate, Dulbecco's phosphate buffered saline (D-PBS), and Hanks' balanced salt solution (HBSS) were purchased from Gibco (Paisley, Scotland). Phorbol 12-myristate 13-acetate (PMA), bovine serum albumin (BSA), fluorescein isothiocyanate (FITC)-labeled BSA, propidium iodide (PI), Hoechst 33258, Triton X-100, ethylenediaminetetraacetic acid (EDTA), paraformaldehyde (PFA; 36% solution), 2-mercaptoethanol (2-ME), trypan blue, horseradish peroxidase (HRP), luminol, cholesterol oxidase from* N. erythropolis *(21.2 U/mL in 1 M ammonium sulfate solution, pH 6), lipoteichoic acid from* Staphylococcus aureus* (LTA), inhibitor of interleukin-1 receptor-associated kinase 1 and 4 (IRAK-1/4 inhibitor, 1-(2-(4-Morpholinyl) ethyl)-2-(3-nitrobenzoylamino) benzimidazole), penicillin/streptomycin solution were from Sigma-Aldrich (St. Louis, MO, USA). Human type AB serum (off-the-clot) and fetal bovine serum (FBS) were purchased from PAA Laboratories (Pasching, Austria). Phycoerythrin (PE)-conjugated mouse IgG2a (*γ*) anti-human TLR2 antibodies (clone TL2.1) and PE-conjugated mouse IgG2a (*γ*) isotype control were from Imgenex (San Diego, CA, USA). PE-conjugated mouse IgG1 (*γ*) anti-human CR3 (CD11b/Mac-1, integrin *α*M chain; clone ICRF44), PE-conjugated mouse IgG1 (*γ*) isotype control, PE-conjugated mouse antiphosphorylated (pT180/pY182) p38 mitogen-activated protein kinase (MAPK) monoclonal antibody (mAb; clone 36/p38), mouse antitotal-p38 MAPK IgG1, PE-conjugated mouse antiphosphorylated (pT202/pY204) extracellular signal-regulated kinase (ERK1/2) mAb (clone 20A), BD Phosflow Alexa Fluor 488-conjugated mouse antitotal-ERK2 mAb (clone G 263-7), and FITC-conjugated goat anti-mouse IgG/IgM were from BD Biosciences (Franklin Lakes, NJ, USA). Donkey anti-rabbit IgG R-PE-conjugated F (ab′)_2_ fragment (H + L) with minimal cross-reactivity to human and mouse serum proteins was from Jackson ImmunoResearch (West Grove, PA, USA). The rabbit anti-ChoD (*N. erythropolis*) polyclonal antibody was from LifeSpan Biosciences (Seattle, WA, USA). The mouse anti-LTA mAb was obtained from ThermoFisher Scientific (Rockford, IL, USA). FluoReporter FITC Protein Labeling Kit and CellTracker Red CMTPX were from Molecular Probes (Carlsbad, CA, USA). Mowiol was from Calbiochem/Merck Chemical Co. (Darmstadt, Germany). The Quantikine Human IL-10 Immunoassay was purchased from R&D Systems Inc. (Minneapolis, MN, USA).

### 2.2. Cell Culture

The THP-1 monocyte-macrophage cell line (TIB-202; American Type Culture Collection, Manassas, VA, USA) was cultured in RPMI-1640 culture medium (CM) supplemented with 1 mM sodium pyruvate, 10% FBS, 0.05 mM 2-ME, 100 U/mL penicillin, and 100 mg/mL streptomycin at 37°C in a 5% CO_2_ atmosphere. Cells were cultured with 20 ng/mL PMA in CM for 24 h to allow for differentiation of THP-1 monocytes into THP-1-derived macrophages, as described previously [15]. After incubation with PMA, CM was removed and macrophages were treated with different concentrations of ChoD for limited periods, as indicated in figures. In all experiments, cells not treated with ChoD were considered control cells, unless otherwise indicated.

### 2.3. Propidium Iodide (PI) Assays of Cell Viability

THP-1-derived macrophages cultured in 24-well plates (1 × 10^6^ cells/well) were treated with ChoD in CM (0.01–0.5 U/mL), or left untreated, for 24 h at 37°C in a 5% CO_2_ atmosphere. Cells were then detached from plates by treatment with trypsin-EDTA for 5 min. Cells were washed once by centrifugation (300 ×g, 5 min) with RPMI-1640 medium containing 10% FBS, suspended in D-PBS/1% FBS (1 × 10^6^ cells/mL) and incubated with PI (2 *μ*g/mL) for 30 min in the dark at room temperature. Dead (necrotic) cells were stained with PI, which penetrates impaired cellular membranes and incorporates into DNA. Samples were analyzed by flow cytometry using an LSR II BD instrument (Becton Dickinson, Franklin Lakes, NJ, USA), with cell acquisition controlled by BD FACSDiva software; 0.8 × 10^4^ cells per sample were counted. Data were analyzed with WinMDI 2.8 flow cytometry application software. The viability of cells treated with ChoD relative to untreated cells was determined on the basis of PI fluorescence intensity. The results were presented as the percentage of PI-positive cells.

### 2.4. ROS Production Assay

ROS production was measured using a luminol-enhanced chemiluminescence method (L-CL). THP-1 monocytes (1 × 10^5^ cells/well) were distributed into 96-well white plates and differentiated into macrophages, as described above. Cells were then treated with ChoD in CM (0.01 to 0.5 U/mL) for 1, 2, and 24 h or were left untreated (37°C, 5% CO_2_). In some experiments, cells were pretreated with IRAK1/4 inhibitor (1 *μ*M, 1 h, 37°C, 5% CO_2_) and then treated with ChoD, as described above. After incubation, supernatants were removed and cells were gently washed with warm HBSS, 1 *μ*g/mL of PMA in HBSS (to initiate ROS production); 1 mM luminol and 40 U of HRP (to enhance chemiluminescence) were added to cells and L-CL was recorded over 4 h at 5 min intervals in a Fluoroskan Ascent FL fluorometer (Labsystems, Helsinki, Finland). CL intensity was given in relative light units (RLU). The area under the curve of L-CL versus assay time (total RLU of integrated signals) was calculated.

### 2.5. IL-10 Production

THP-1-derived macrophages seeded on 24-well plates (1 × 10^6^ cells/well) were cultured for 24 h with ChoD in CM (0.001 to 0.5 U/mL) or left untreated (37°C, 5% CO_2_). The culture medium was then collected and centrifuged (12000 ×g, 5 min), and the resulting supernatants were assayed for IL-10 using Quantikine ELISA kits, according to manufacturer's procedure. The sensitivity of the IL-10 assay was 3.9 pg/mL. The value for untreated cells was 0.1 ± 0.1 pg IL-10/mL/10^6^ cells.

### 2.6. Expression of TLR2 and CR3 on Macrophages

THP-1-derived macrophages seeded on 24-well plates (1 × 10^6^ cells/well) were treated with ChoD in CM (0.01 to 0.5 U/mL) for 1, 2, and 24 h, or left untreated (37°C, 5% CO_2_). Cells were then detached from plates by treatment with trypsin-EDTA for 5 min (37°C, 5% CO_2_), after which trypsin was neutralized by adding an equal volume of RPMI-1640 medium containing 10% FBS. Cells were washed by centrifugation (300 ×g, 5 min) and suspended in D-PBS/1% FBS. The viability of collected cells, determined using the trypan blue exclusion method, was ~95%. Cells were incubated with 10% human AB serum in D-PBS for 20 min at room temperature in order to prevent nonspecific antibody binding to Fc receptors. Thereafter, cells were washed twice with D-PBS/1% FBS and stained with PE-conjugated mouse anti-TLR2 (10 *μ*g/mL) or anti-CR3 (5 *μ*g/mL) mAbs, or appropriate isotype controls, for 30 min at 4°C. After staining, cells were washed twice with D-PBS/1% FBS, fixed with 1% PFA in D-PBS/1% FBS for 30 min at room temperature, washed once and suspended in D-PBS/1% FBS, and examined using an LSR II BD flow cytometer as described above. The results were presented as median fluorescence intensity (MFI). Control experiments showed that ChoD did not interfere with the binding of anti-TLR2 or anti-CR3 antibodies to macrophages.

### 2.7. The p38 MAPK and ERK1/2 Phosphorylation Assays

The phosphorylation of p38 MAPK and ERK on tyrosine and threonine residues was measured using a flow cytometry-based method. THP-1-derived macrophages seeded on 24-well plates (1 × 10^6^ cells/well) were treated with different concentrations of ChoD in CM (24 h, 37°C, 5% CO_2_, 0.01 to 0.5 U/mL), or were left untreated, for 24 h (37°C, 5% CO_2_). Macrophages were then gently washed and detached from plates by incubating for 30 min on ice with 1 mL cold D-PBS/2 mM EDTA with brief pipetting. Cells were then immediately fixed with 4% PFA for 10 min, washed twice with D-PBS, and permeabilized by incubating with ice-cold 80% methanol (final concentration) for at least 1 h on ice. After permeabilization, cells were washed three times with D-PBS, blocked with 10% FBS in D-PBS for 30 min at room temperature, and stained with the following antibodies: PE-conjugated mouse antiphospho-p38 MAPK (pT180/pY182), PE-conjugated mouse antiphospho-ERK1/2 (pT202/pY204) or Alexa Fluor 488-conjugated mouse anti-total-ERK2 (20 *μ*L/mL for all antibodies) for 1 h in the dark. For total p38 MAPK detection, mouse anti-p38 MAPK IgG1 or appropriate IgG1 isotype control (20 *μ*g/mL) was added to fixed and permeabilized cells, incubated for 1 h at room temperature, washed, and stained with FITC-conjugated goat anti-mouse secondary antibody (10 *μ*g/mL) for 30 min at 4°C. After staining, cells were washed twice with D-PBS/1% FBS, suspended in D-PBS, and examined for fluorescence by flow cytometry, as described above. The results were presented as MFI of cells.

### 2.8. Measurement of ChoD Binding to Macrophages Using Flow Cytometry

Binding of ChoD to THP-1-derived macrophages was determined by flow cytometry using FITC-labeled ChoD. ChoD was conjugated with FITC dye using a FluoReporter FITC Protein Labeling Kit. Recovery of protein was ~80%, and the labeling ratio was 1 : 1.5 (mole : mole, ChoD : FITC), as determined by the Proteins and Labels module in the NanoDrop 2000c/2000 UV-Vis Spectrophotometer (Thermo Scientific, Fremont, CA, USA). The amount of FITC-ChoD used in these studies was equivalent to 0.5 U/mL of unlabeled enzyme (24 *μ*g/mL). FITC-BSA at a concentration of 24 *μ*g/mL was used as a control. Control experiments showed that ChoD activity was unaffected by labeling, with both FITC-labeled and unlabeled ChoD influencing TLR2 and CR3 expression to the same extent (data not shown). BSA and FITC-BSA, used as references, did not affect macrophage activity (data not shown).

Macrophages seeded on 24-well plates (1 × 10^6^ cells/well) were incubated with FITC-ChoD and reference FITC-BSA (24 *μ*g/mL), or left untreated (control cells for measuring autofluorescence), for 24 h (37°C/5% CO_2_). After incubation, cells were washed, detached from plates by trypsinization, and immediately fixed by incubating with 1% PFA in D-PBS/1% FBS for 30 min at room temperature. The extracellular fluorescence of FITC-ChoD was determined by trypan blue (0.5%) quenching. Fluorescence of cells was examined by flow cytometry, as described above.

Binding of ChoD to THP-1-derived macrophages was also determined by staining of ChoD with rabbit anti-ChoD (*N. erythropolis*) polyclonal antibody and R-PE-conjugated donkey anti-rabbit secondary antibody. For intracellular staining, cells treated with ChoD for 24 h (37°C, 5% CO_2_) were washed with D-PBS and permeabilized with ice-cold 80% methanol for at least 1 h on ice. After permeabilization, cells were washed with D-PBS, blocked with 10% FBS in D-PBS for 30 min at room temperature, and antibodies were added. After staining, cells were extensively washed with D-PBS/1% FBS, suspended in D-PBS, and examined by flow cytometry, as described above. The results were presented as MFI of cells.

### 2.9. Measurement of ChoD Binding to Macrophages Using Fluorescence Microscopy

Cellular localization of ChoD in macrophages following a 24 h incubation was examined by confocal microscopy using FITC-labeled ChoD. THP-1 monocytes were distributed on glass 8-well Nunc Lab-Tek II Chamber Slides (NUNC, Denmark) at a density of 1 × 10^5^ cells per well and cultivated in CM with 20 ng/mL PMA to differentiate monocytes into macrophages, as described above. Macrophages were gently washed with D-PBS/10% FBS and incubated with FITC-ChoD, or left untreated (for assessment of background fluorescence), for 24 h (37°C/5% CO_2_). The amount of FITC-ChoD used in these studies was equivalent to 0.5 U/mL of unlabeled enzyme (24 *μ*g/mL). FITC-BSA at a concentration of 24 *μ*g/mL was used as a control. After incubation of cells with FITC-ChoD or FITC-BSA, all subsequent steps were carried out at room temperature. Cells were gently washed once with D-PBS/10% FBS, twice with D-PBS, and stained with CellTracker Red CMTPX (cytoplasm-selective fluorescent dye) at a concentration of 5 *μ*M in D-PBS for 30 min in the dark. Cell nuclei were stained with 5 *μ*g/mL Hoechst 33258 in D-PBS for 15 min. After staining procedures, cells were gently washed once with D-PBS/10% FBS, twice with D-PBS and then fixed in 4% PFA for 10 min. Slides were then rinsed twice with D-PBS and sterile water and mounted with Mowiol. Cells were visualized using a confocal microscope (Nikon D-Eclipse C1; Nikon, Tokyo, Japan) with a 40x objective and analyzed with EZ-C1 version 3.6 software. The different fluorophores were detected using appropriate filter sets. The pixel resolution of digital images acquired with the 40x objective was 1024 × 1024. Triplicate slides were prepared for each experiment. The same cells were analyzed in parallel by flow cytometry, as described above.

In another set of experiments, THP-1-derived macrophages, seeded on Permanox 8-well Nunc Lab-Tek Chamber Slides (1 × 10^5^ cells/well), were incubated briefly (30 min) with ChoD (0.5–1 U/mL in D-PBS/5% FBS) at room temperature. The TLR2 agonist, LTA (100 *μ*g/mL in D-PBS/5% FBS), was added to the cells as a marker of TLR2 ligand-binding sites. Incubation times and temperatures were selected to maintain conditions appropriate for the binding of ChoD and LTA to cells while preventing macrophage activation. After incubation, cells were gently washed three times with 5% FBS in D-PBS and stained with primary antibodies against* N. erythropolis* ChoD (rabbit polyclonal antibody, 20 *μ*g/100 *μ*L) and LTA (mouse mAb, 1 *μ*g/100 *μ*L) in D-PBS for 30 min. Nonspecific binding was blocked by incubating with 10% FBS in D-PBS for 20 min. Samples were then gently washed three times with 5% FBS in D-PBS and incubated with FITC-conjugated anti-mouse or PE-conjugated anti-rabbit secondary antibodies (0.5 *μ*g/100 *μ*L) for 20 min in the dark. Thereafter, the slides were washed twice with D-PBS, fixed with 4% PFA for 10 min, and rinsed twice with D-PBS and sterile water. Slides were mounted with Mowiol and allowed to set overnight at room temperature. Cells were visualized under a Nikon Eclipse TE 2000-U microscope (Nikon Plan oil 100x/1.25 objective) equipped with blue excitation bandpass B-2E/C and green excitation G-2A filters and a DS-U2 digital color camera (Nikon, Japan), using equal exposure times and gain parameters. Background fluorescence of nonspecific antibodies and the spectral overlap between channels was negligible (i.e., below threshold). The pixel resolution of acquired digital images was 2560 × 1920.

Microscopic images were analyzed using NIS Elements AR Analysis 3.2 software after preprocessing by deconvolution (Deconvolution Lab plug-in in the software ImageJ 1.47 v). Threshold fluorescence was subtracted from image fluorescence separately for each channel. Mean background intensity levels were calculated from image regions outside of areas occupied by cells. Pixel colocalization was displayed and analyzed using the colocalization module and pixel classifier tools in NIS Elements AR Analysis 3.2 software.

### 2.10. Examination of ChoD and LTA Competitive Binding to Cells

THP-1-derived macrophages in suspension (1 × 10^5^ cells/well) were first preincubated with ChoD (0.5 or 1 U/mL in D-PBS/5% FBS) for 30 min at room temperature and then washed and subsequently incubated with LTA (10 or 100 *μ*g/mL in D-PBS/5% FBS) for 30 min. Thereafter, cells were gently washed two times with 10% FBS in D-PBS and stained with rabbit polyclonal anti-ChoD (20 *μ*g/100 *μ*L) and mouse monoclonal anti-LTA (1 *μ*g/100 *μ*L) primary antibodies in D-PBS for 30 min and subsequently with FITC-conjugated anti-mouse or PE-conjugated anti-rabbit secondary antibodies (0.5 *μ*g/100 *μ*L) for 20 min. In secondary experiments, cells were first preincubated with LTA (10 or 100 *μ*g/mL), then subsequently incubated with ChoD (0.5 or 1 U/mL) and stained as described above. The samples were then washed twice with D-PBS, fixed with 1% PFA, and analyzed by flow cytometry.

### 2.11. Statistical Analysis

Statistical significance was verified using nonparametric Wilcoxon's signed-rank test, unless otherwise indicated. The Statistica software package, version 10 (StatSoft, Poland), was used for statistical calculations. Statistical significance was set at *P* < 0.05.

## 3. Results

### 3.1. ChoD Treatment Decreases the Levels of TLR2 and CR3 Receptors on THP-1-Derived Macrophages

**Figure 1:**
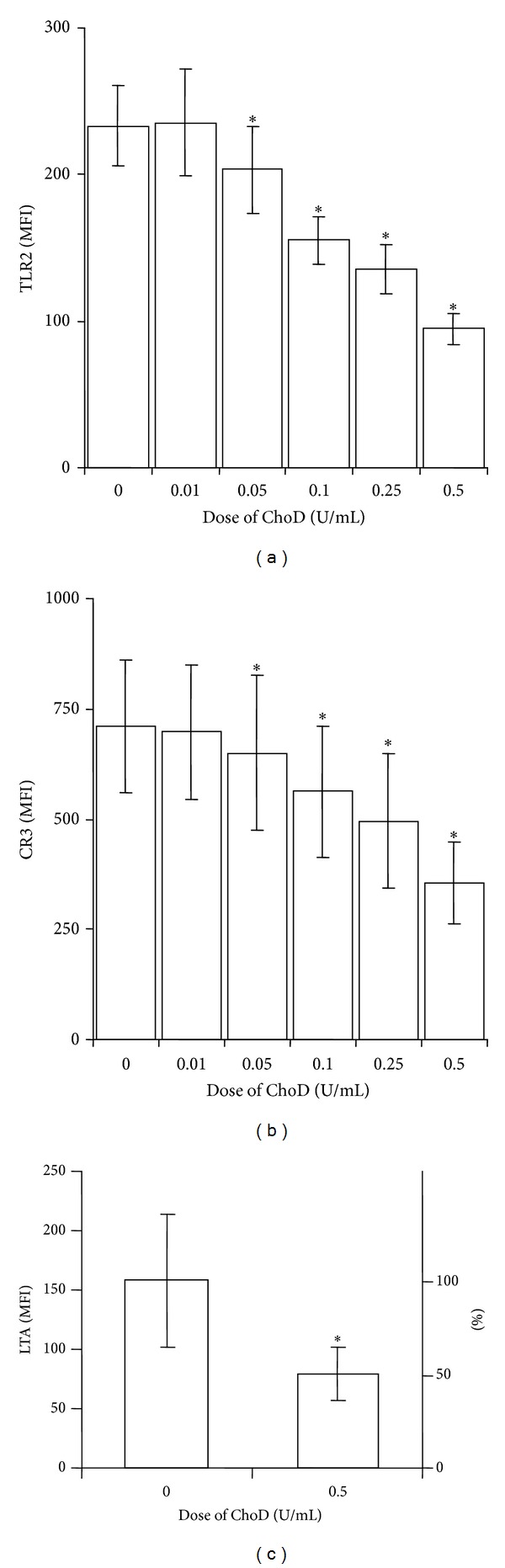
The effect of ChoD on TLR-2 and CR3 expression on THP-1-derived macrophages. TLR2 (a) and CR3 (b) expression on cells treated with ChoD (0.01–0.5 U/mL) for 24 h. Data are means ± SEM of MFI values from 5-6 separate experiments (*P < 0.05 for macrophages + ChoD* versus* macrophages). (c) LTA (100 *μ*g/mL, 30 min) binding to macrophages pretreated with ChoD (0.5 U/mL) for 24 h. After treatment with ChoD and LTA, macrophages were incubated with anti-LTA antibodies and secondary FITC-conjugated antibodies and then analyzed using flow cytometry. Data are expressed as means ± SEM of MFI values from seven independent experiments (*P < 0.05 for macrophages + ChoD + LTA* versus* macrophages + LTA). Percentage values relative to control (100%) are shown on the right axis.

We have previously shown that intact choD gene is indispensable for the virulence of tubercle bacilli [9, 10]; however, it is not essential for cholesterol degradation [6]. To evaluate whether ChoD* per se* affects human macrophages (THP-1 cells), we used commercially available ChoD from* N. erythropolis,* which is highly similar to its ortholog in Mtb. An initial evaluation of ChoD toxicity showed that ChoD was not cytotoxic for macrophages at concentrations up to 0.5 U/mL; at a higher concentration (1 U/mL) ChoD was not toxic at shorter incubation times (30 min, 1 h and 2 h; data not shown) but caused necrotic cell death of ~40% of cells with a longer incubation time (24 h), as determined by PI staining (data not shown). We then assessed whether ChoD affected the expression of pattern-recognition receptors. Treatment of THP-1-derived macrophages with ChoD (0.5 U/mL) for 24 h downregulated the surface expression of TLR2 and CR3 (Figures 1(a) and 1(b)), decreasing their levels by 2.5- and 2-fold, respectively, as assessed by flow cytometry using specific antibody staining. This treatment was associated with a remarkable decrease in TLR2 function, as evidenced by a decrease in LTA binding to these cells (Figure 1(c)). However, shorter incubations (1 and 2 h) of macrophages with ChoD (0.1 and 0.5 U/mL) did not affect TLR2 levels (data not shown). Moreover we have found that the TLR4 expression on THP-1-derived macrophages was undetectable by flow cytometry method, and the treatment of cells with ChoD did not induce the TLR4 expression on cellular membrane (data not shown).

### 3.2. ChoD Co-Localizes to TLR2 but Not to CR3 on THP-1-Derived Macrophages

**Figure 2:**
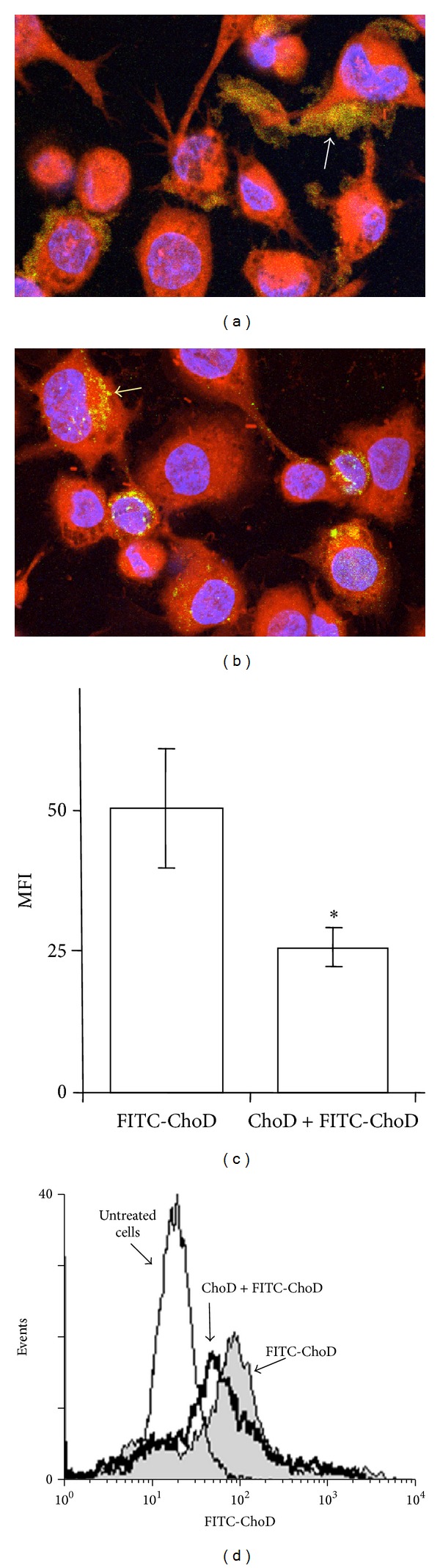
FITC-ChoD binding to THP-1-derived macrophages determined by confocal microscopy and flow cytometry. Macrophages were incubated FITC-ChoD (24 *μ*g/mL, equivalent to 0.5 U/mL) (a) or FITC-BSA (24 *μ*g/mL) as a reference protein (b) and then stained with CellTrackerRed (red fluorescence, cytoplasm) and Hoechst 33258 (blue fluorescence, nuclei). Cells were analyzed by confocal microscopy (40x objective). Extracellular localization of FITC-ChoD is marked by white arrow (a). Intracellular localization of FITC-BSA is marked by yellow arrow (b). (c, d) Binding of FITC-labeled ChoD to macrophage membranes before and after incubation with unlabeled ChoD. Cells incubated with or without ChoD (0.5 U/mL) for 30 min were subsequently incubated with FITC-ChoD (24 *μ*g/mL, equivalent to 0.5 U/mL) for 1 h. Fluorescence was measured by flow cytometry and expressed as MFI. The fluorescence intensity of cells not treated with FITC-ChoD was defined as autofluorescence and was subtracted from the fluorescence values of corresponding FITC trials. Data are means ± SEM of MFI values from three independent experiments (c). A representative histogram shows the fluorescence of (1) cells incubated with FITC-ChoD, (2) cells preincubated with unlabeled ChoD and then with FITC-ChoD, and (3) autofluorescence of untreated cells (d).

**Figure 3:**
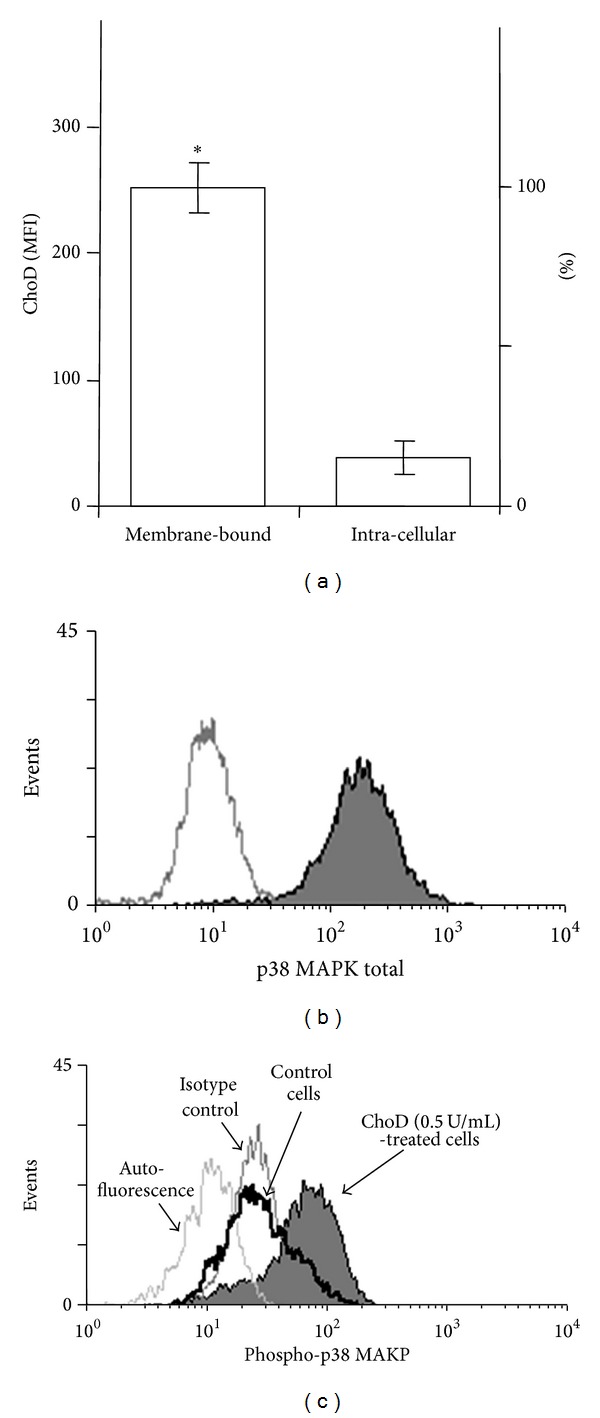
Membrane and intracellular location of ChoD in THP-1-derived macrophages. Macrophages were incubated with ChoD (0.5 U/mL) for 24 h and stained with anti-ChoD primary and PE-conjugated secondary antibodies. ChoD was detected on intact cells (membrane location) and in permeabilized cells (intracellular location). Data are means ± SEM of MFI values from six separate experiments (*P < 0.05) (a). The representative histograms show the fluorescence intensities of intact (b) and permeabilized cells (c).

**Figure 4:**
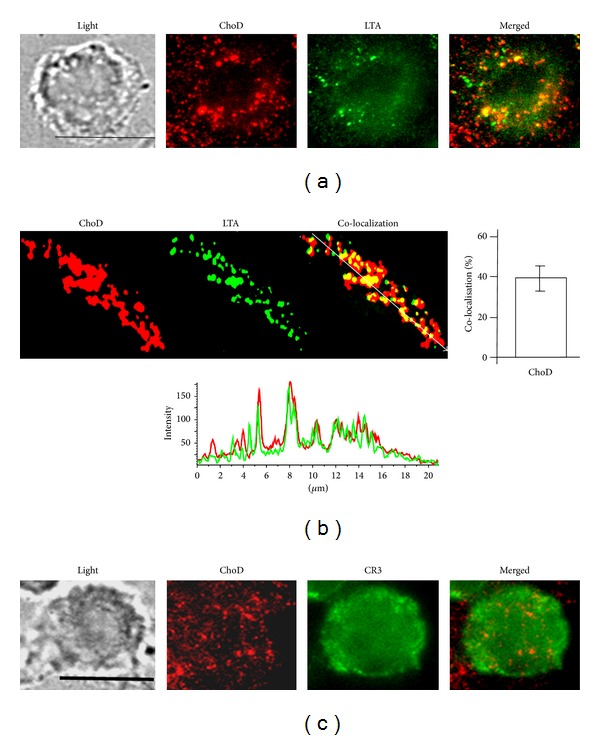
Determination of ChoD binding to LTA-binding sites in THP-1-derived macrophages. Macrophages were incubated with ChoD (0.5 U/mL) and LTA (100 *μ*g/mL) for 30 min. After fixation with 4% PFA, cells were evaluated using a Nikon Eclipse TE 2000-U microscope. At least 10 fields were analyzed per slide (100x objective; scale bar = 10 *μ*m). Three independent experiments were carried out. (a) Fluorescence of double-labeled macrophages after incubation with ChoD (PE-antibody staining, red) and LTA (FITC-antibody staining, green). The merged image shows extensive overlay of areas positive for ChoD and LTA (yellow). (b) Enlarged fluorescence images of the representative region of colocalization. Individual markers are shown in red (ChoD) and green (LTA). Merged image showing colocalization of LTA and ChoD staining (yellow) is shown in the panel at right. Graph at right shows mean value ± SEM of the percent of ChoD areas that colocalize with LTA (six individual regions of evident colocalization per slide; three independent experiments). Graph below shows fluorescence-intensity profiles of ChoD (red) and LTA (green) in a cross-section (denoted by an arrow on the merged image), demonstrating excellent colocalization. (c) Representative image of cell shows lack of colocalization of ChoD and CR3.

**Figure 5:**
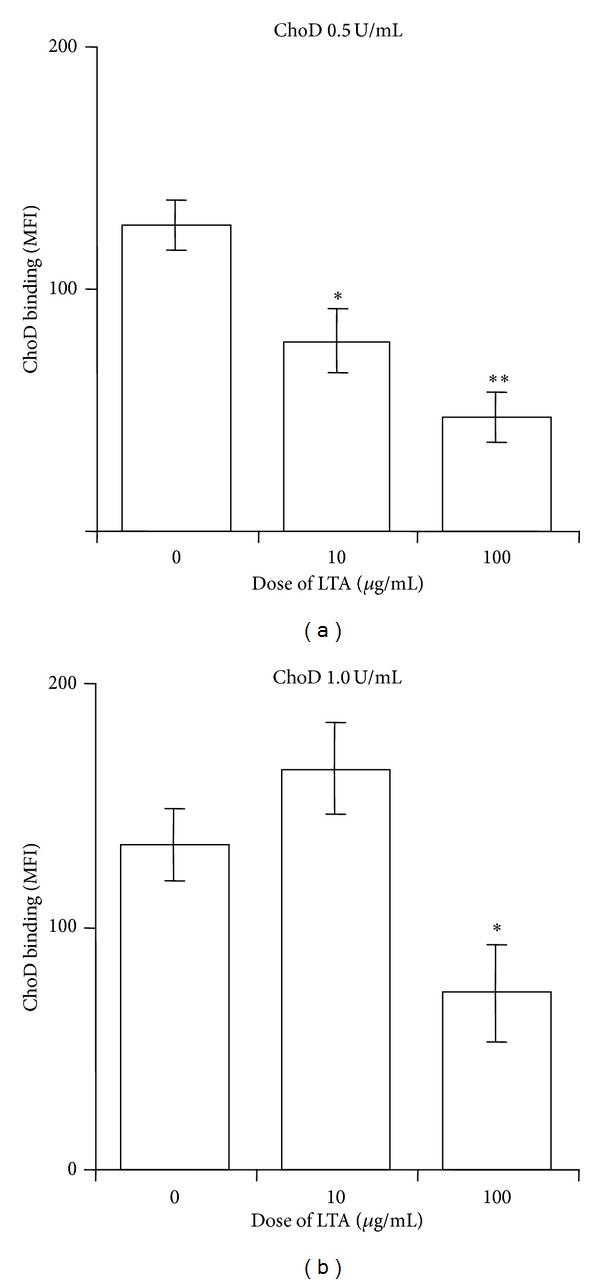
Competition of ChoD and LTA for binding sites on THP-1-derived macrophages. Macrophages were preincubated with or without LTA (10 or 100 *μ*g/mL) and then treated with ChoD (0.5 U/mL or 1 U/mL, (a) or (b), resp.) for 30 min at room temperature. After treatment, cells were stained as described in Section 2, and MFI values were measured by flow cytometry. Cells that were not treated with LTA or ChoD, but were stained with antibodies, were used for examination of nonspecific binding. The values for nonspecific binding were subtracted from the corresponding sample values. Data are means ± SEM of MFI values from three separate experiments (*P < 0.05, **P < 0.01, for LTA-untreated macrophages* versus* LTA-treated macrophages; Student *t*-test).

Next we analyzed whether ChoD binds to macrophages using FITC-labeled ChoD in conjunction with confocal microscopy. These analyses revealed that ChoD was mainly located at the cellular membrane of macrophages (Figure 2(a)). Using the pixel classifier tool in Nis Elements software, we found that the green fluorescence of FITC-ChoD and red fluorescence of the cytoplasm were located independently (i.e. nonoverlapping). In contrast, a control experiment showed that FITC-BSA was mainly located in the cytoplasm, as evidenced by the yellow color in merged images (Figure 2(b)). Flow cytometry also showed that FITC-ChoD was associated with the membrane of macrophages and that preincubation with unlabeled enzyme greatly reduced FITC-ChoD binding to cells (Figures 2(c) and 2(d)). Flow cytometry using specific anti-ChoD antibodies applied to intact cells (membrane location) and cells permeabilized with methanol (intracellular location) further confirmed the cell surface localization of ChoD (Figures 3(a), 3(b), and 3(c)). Quantification of these results showed that approximately 85% of ChoD was associated with the cellular membrane and only about 15% was located inside the cell. Next, we used epifluorescence microscopy to examine binding of ChoD to TLR2 receptors on THP-1-derived macrophages; LTA was used as a specific TLR2 ligand. ChoD (red) and LTA (green) were detected in cells by specific fluorescent antibodies (Figures 4(a) and 4(b)). A statistical analysis of six selected regions from three independent images showed that up to 40% ± 6% of ChoD areas colocalized with LTA binding sites (Figure 4(b)). Cross-sections of analyzed images revealed an overlap of ChoD and LTA fluorescence intensity in colocalization areas (Figure 4(b)). These data were supported by a laser-scanning cytometry analysis of a large number of regions of LTA and ChoD binding to macrophages (up to 6000 cells analyzed, in duplicate). An analysis of these data confirmed that 42% of analyzed regions exhibited cooccurrence of ChoD and LTA. Moreover, the fluorescence intensities of ChoD and LTA regions were highly correlated (see Supplementary Figure S1 in Supplementary material available online at http://dx.doi.org/10.1155/2014/498395). Notably, microscopic examinations revealed no colocalization of ChoD with CR3 (Figure 4(c)). To assess ChoD and LTA competition for the TLR2 binding site, we performed experiments in which macrophages were consecutively treated with both compounds for a short time period (30 min) at room temperature, under conditions that allowed for optimal binding of ChoD and LTA without activating macrophages. Neither ChoD nor LTA interfered with antibody staining of receptors (data not shown). Flow cytometry revealed that preincubation with LTA decreased ChoD binding to macrophages in a concentration-dependent manner (Figures 5(a) and 5(b)), reducing ChoD binding by more than 2.5-fold at a concentration of 100 *μ*g/mL (Figure 5(a)). The highest dose of ChoD (1 U/mL) overcame the effects of 10 *μ*g/mL LTA, but was unable to surmount the effects of 100 *μ*g/mL LTA, which diminished enzyme binding (Figure 5(b)). In control experiments, pre-incubation of macrophages with fibronectin (CR3 ligand, 20 *μ*g/mL) did not affect ChoD binding to macrophages (data not shown).

### 3.3. ChoD Modulates the Biological Activity of THP-1-Derived Macrophages

**Figure 6:**
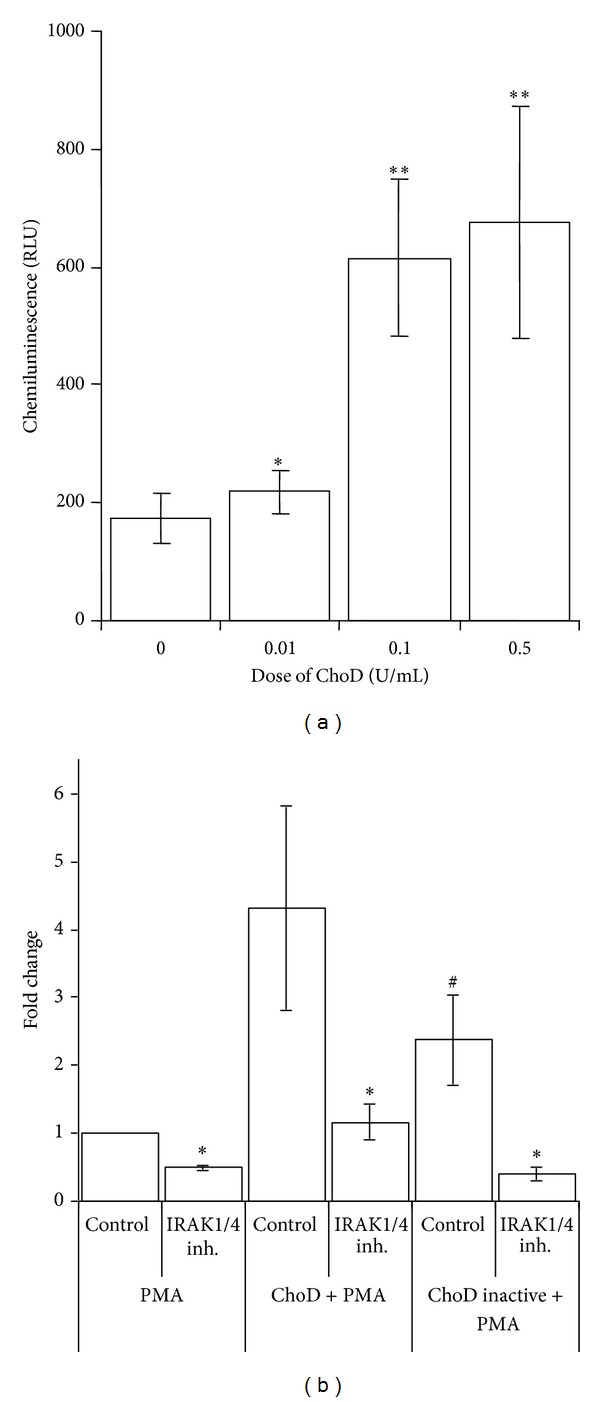
Effect of ChoD on ROS production by THP-1-derived macrophages stimulated with PMA. (a) Macrophages were incubated with ChoD (0.01–1 U/mL) for 24 h; untreated cells were used as controls. ROS production was initiated by exposure to PMA and determined by L-CL assay as described in Section 2. Data are means ± SEM of seven separate experiments (*P < 0.05, **P < 0.01, macrophages + ChoD (0.5 U/mL) + PMA* versus* macrophages + PMA. The RLU values were 26 ± 3 for control cells (no PMA, no ChoD) and 25 ± 4, 24 ± 3, 25 ± 3, and 25 ± 3 for cells treated with ChoD only at concentrations of 0.01, 0.1, 0.5 and 1 U/mL, respectively. (b) Influence of IRAK1/4 inhibitor on the noninactivated and heat-inactivated ChoD effects on ROS production (fold change). Data are means ± SEM of four separate experiments (*P < 0.05 for IRAK1/4 inhibitor* versus *control, ^#^ *P* < 0.05 heat-inactivated ChoD* versus* noninactivated ChoD, Student *t*-test).

**Figure 7:**
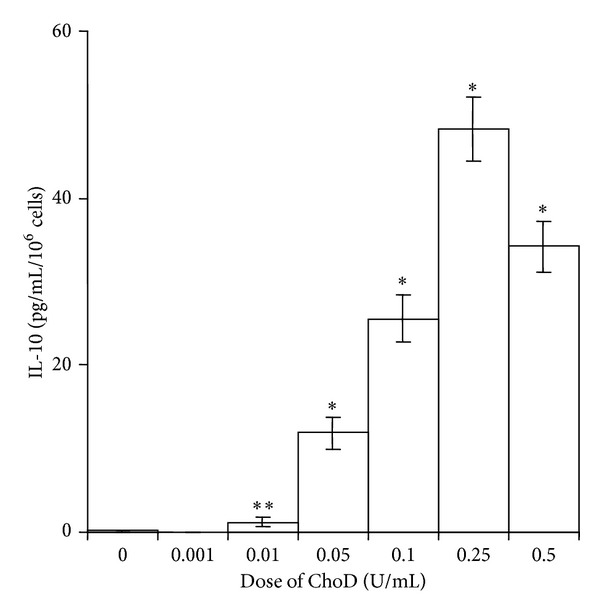
Effects of ChoD on IL-10 release from THP-1-derived macrophages. Macrophages were treated with ChoD (0.001–0.5 U/mL) for 24 h or left untreated. IL-10 was assessed in supernatants collected from cell cultures. The control value was 0.1 ± 0.1 pg/mL. Data are means ± SEM of six separate experiments (*P < 0.05, **P < 0.01 for macrophages + ChoD* versus* macrophages).

Incubation of macrophages with ChoD (0.01–0.5 U/mL) for 1, 2 and 24 h did not influence the basal production of ROS (27 ± 2.3 RLU for 0.5 U/mL ChoD* versus *25 ± 2.5 RLU for untreated cells). However, preincubation of macrophages with ChoD for 24 h resulted in significant, concentration-dependent changes in ROS production in PMA-stimulated macrophages (Figure 6(a)). At a concentration of 0.5 U/mL, ChoD (24 h treatment) increased PMA-stimulated ROS levels ~4-fold (Figure 6). Macrophages preincubated with ChoD at a concentration of 1 U/mL for 24 h did not respond to PMA stimulation (data not shown). Preincubation of macrophages with 1 U/mL of ChoD for a shorter time (1 and 2 h) significantly reduced PMA-induced ROS production (~2-fold decrease, *P* < 0.05), whereas treatment with 0.5 U/mL of ChoD under these conditions had no effect (Figure 6(b)). The ROS production in PMA-stimulated macrophages treated with ChoD (0.5 U/mL) for 24 h) was significantly reduced in cells preincubated with the inhibitor of interleukin-1 receptor-associated kinases 1 and 4 (IRAK1/4) which are functionally associated with TLR signaling (Figure 6(b)). Moreover, the antibodies neutralizing TLR2 signaling pathway caused the decrease of ROS production enhanced by ChoD in PMA-stimulated macrophages. In contrast, an employment of antibodies blocking TLR4 pathway did not affect the ROS production in PMA-stimulated macrophages treated with ChoD (data not shown). What is important, the ROS production was also lowered in macrophages treated with heat-inactivated ChoD compared with cells treated with noninactivated enzyme. The effect of nonenzymatic of heat-inactivated ChoD (Figure 6(b)) was completely abolished by IRAK1/4 inhibitor. Functional studies revealed also that macrophages incubated for 24 h with ChoD (0.001–0.5 U/mL) produced a large amount of IL-10 in a concentration-dependent manner (Figure 7).

**Figure 8:**
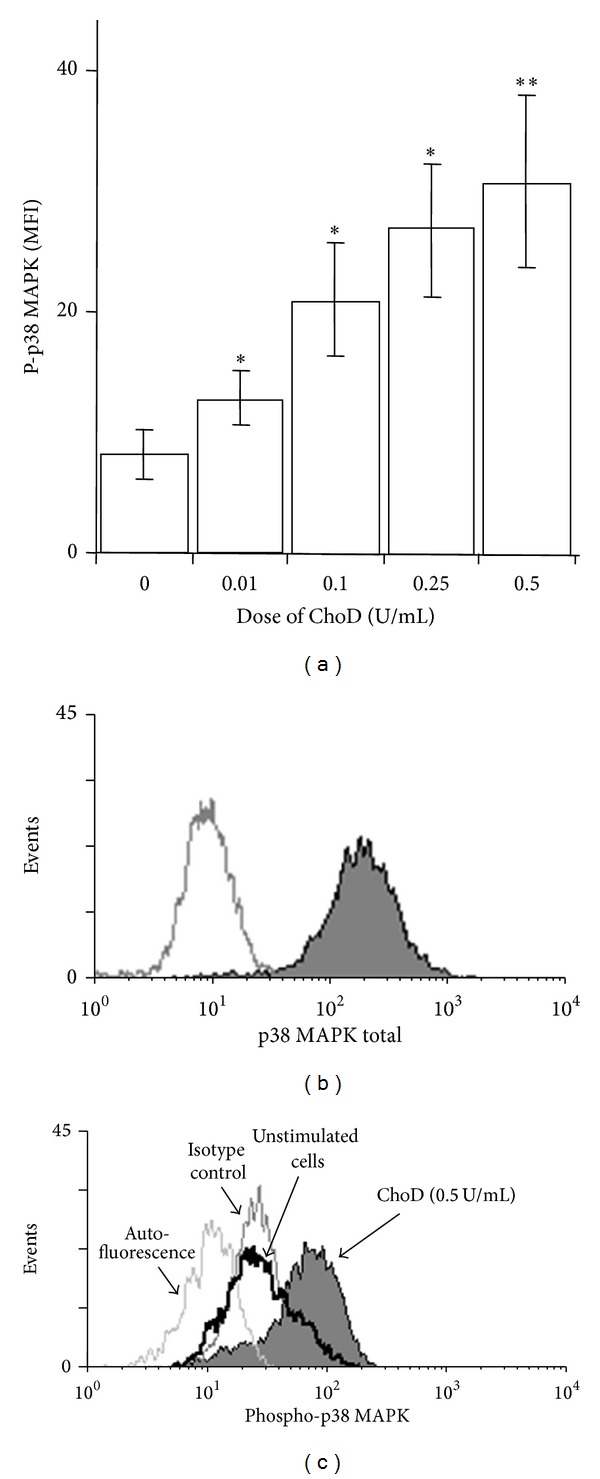
Effect of ChoD on the phosphorylation of p38 MAPK. THP-1-derived macrophages were treated with ChoD (0.01–0.5 U/mL) for 24 h or left untreated (controls). Phosphorylation of kinases was assessed by flow cytometry. Data are expressed as means ± SEM of MFI values from 7-8 independent experiments (*P < 0.05, **P < 0.02 for macrophages + ChoD* versus* macrophages). Representative histograms demonstrate fluorescence intensity of total (b) and phosphorylated p38 MAPK (c). The differences in p38 MAPK phosphorylation in control macrophages as well as macrophages treated with ChoD (0.5 U/mL) are shown.

Finally, the ChoD effects on phosphorylation of MAPKs in macrophages was assessed. These experiments showed that ChoD induced a concentration-dependent increase in the phosphorylation of p38 MAPK in macrophages (Figure 8). However, the phosphorylation of ERK1/2 remained unchanged after treatment with ChoD.

## 4. Discussion 

We have previously shown that a Mtb mutant unable to synthesize ChoD exhibits attenuated growth and survival in human macrophages [10] and in a mouse experimental model of tuberculosis [9]. This attenuated pathogenesis was not due to a failure to degrade cholesterol or consequent effects on the availability of cholesterol as a carbon and energy source since ChoD enzymatic activity was not essential for this process [6, 10]. Here we sought to assess the effect of ChoD on the biological activity of human macrophages* in vitro*. For this purpose, we used a commercially available ChoD from* N. erythropolis, *which is highly similar to its ortholog in Mtb.

We found that ChoD colocalizes with the TLR2 receptor on human macrophages, demonstrating that ChoD targets sites bound by the TLR2 receptor ligand, LTA, in the macrophage membrane and that ChoD and LTA are located in bordering and partly overlapping clusters. Confocal microscopy and LSCR data revealed that about 40% of ChoD binding regions were contained within the TLR2 region, indicating that TLR2 binding sites, or sites at least within their proximity, can be occupied by bacterial ChoD. It has been found that leucine-rich repeat motifs in TLRs recognize a broad variety of agonists, and ligand-interaction sites on TLRs can be the same or different [16]. Our data may indicate that TLR2 is involved in the recognition of ChoD and/or is a common element in the binding of both LTA and ChoD to macrophages. In contrast to TLR2, CR3 was not a target of ChoD binding, suggesting that ChoD might interact selectively with TLR2. Treatment of THP1 cells for 24 h with 0.5 U/mL ChoD decreased the level of both TLR2 and CR3 receptors. This effect was not observed with briefer exposures or lower concentrations of ChoD. It may be that high levels of ChoD indirectly affect receptors by altering lipid rafts in the macrophage membrane, consistent with a previous report describing the decomposition of lipids rafts by ChoD [11]. We also observed that macrophages treated with ChoD bound only half the amount of LTA as untreated cells, indicating that ChoD may remodel the cellular membrane, altering its composition and thereby affecting the expression and function of receptors [11]. Alternatively, pretreatment of macrophages with ChoD may produce tolerance to subsequent treatment with LTA, as suggested by the previous observation that ligands that bind and activate TLRs reduce the sensitivity of these receptors for binding other ligands [17].

In this study, we demonstrated a direct modulating effect of ChoD on the activity of macrophages including stimulation of IL-10 production in resting macrophages and enhancement of ROS production in macrophages stimulated with PMA. A number of stimuli act through the activation of various protein kinases, including MAPKs such as ERK1/2 and p38 MAPK [18–20]. We have shown that ChoD increases the activity of p38 MAPK signaling pathway (by its phosphorylation) in macrophages which then can enhance susceptibility of these cells to stimulation by PMA. One of the more important activities of macrophages is their ability to generate of ROS which is essential for the protection of a host against the invasion of pathogenic bacteria. The priming effect of ChoD on ROS production, shown in our study, was reduced twice through a turning off its enzymatic activity. Furthermore, the effect of heat-inactivating ChoD on ROS production was completely abolished by inhibiting of kinases IRAK1/4. Moreover, the inhibition of IRAK1/4 also significantly reduced the overall effect of a noninactivated enzyme. We found that a direct interaction of the enzyme with TLR2 receptor activates an intracellular signal, which is of functional importance for macrophages.

It is reasonable to suppose that the immunosuppressive activity of ChoD is related to its ability to modify cholesterol in the cellular membrane. Our results support this hypothesis, since, as noted above, ChoD is located at the macrophage cell membrane, which is rich in cholesterol, rather than inside the cell. Therefore, it is likely that the macrophage membrane is the main target for the binding and action of ChoD. It has also been documented by others [21] that intact cholesterol in the monocyte membrane is required for activation of intracellular signaling proteins (e.g., p38 MAPK) in these cells. We found that macrophages treated with ChoD release significant amounts of the anti-inflammatory cytokine IL-10, which inhibits the synthesis of proinflammatory cytokines such as interferon-*γ*, tumor necrosis factor-*α*, IL-2, and IL-1. These latter factors, in turn, are involved in the development of acquired immunity, which is responsible for the eradication of intracellular pathogens [22, 23]. It is worth noting that IL-10 induction was observed with as little as 0.1 U/mL of ChoD, suggesting that the immunosuppressive effect of ChoD could have real biological significance during infection of tubercle bacilli.

A well-known characteristic of macrophages is their ability to acquire a distinct phenotype in response to exposure to various stimuli. Upon stimulation, macrophages undergo a dynamic change in activation status, from classical activated M1 macrophages (e.g., stimulated with TLR ligands) to alternatively activated M2 cells (e.g., stimulated with IL-4, IL-13, and IL-10). The M1 phenotype produces reactive oxygen and nitrogen species, proinflammatory cytokines, and possesses antimicrobial properties. In contrast, M2 macrophages are involved in fighting inflammation and repairing tissue and possess efficient phagocytic activity [24–26]. However, it should also be emphasized that ROS play an important role in the regulation of macrophages activity and switching the proinflammatory to the anti-inflammatory phenotype of macrophages responsible for the production of immunosuppressive cytokines, such as IL-10, that protect the host against inappropriate or excessive inflammatory responses [22, 27]. 

In summary, our results show that extracellular bacterial ChoD exerts diverse effects on the functional activity of macrophages. On the one hand, bacterial ChoD preactivates macrophages to promote ROS production—a proinflammatory effect; but on the other hand, it reduces TLR2 and CR3 expression levels and induces production of anti-inflammatory IL10. Activation of the p38 MAPK signaling pathway was noted in ChoD-treated macrophages. Our results also revealed that ChoD is mainly located in the cell membrane and competes with LTA for TLR2 binding sites. We speculate that binding of ChoD to TLR2 is an important aspect of macrophage function and may determine the virulence of bacteria that produce that enzyme. However, the suggestion that ChoD affects TLR2 signaling pathways in macrophages through a direct interaction with the receptor requires future studies. Since ChoD plays an important role in bacterial pathogenicity (e.g., Mtb), studies of the direct influence of this enzyme on the key element of innate immunity could facilitate the search for new therapeutic agents for possible future clinical use.

## Supplementary Material

Laser-scanning cytometry (LSC) analysis: THP-1-derived macrophages were incubated briefly (30 min) with ChoD (0.5 U/ml) and LTA (100 *μ*g/ml) at room temperature. After incubation, cells were gently washed three times with 5% FBS in D-PBS and stained with primary antibodies against N. erythropolis ChoD (rabbit polyclonal antibody, 20 *μ*g/100 *μ*l) and LTA (mouse mAb, 1 *μ*g/100 *μ*l) in D-PBS for 30 min at room temperature. Non-specific binding was blocked by incubating with 10% FBS in D-PBS for 20 min. Samples were then gently washed three times with 5% FBS in D-PBS and incubated with FITC-conjugated anti-mouse or PE-conjugated anti-rabbit secondary antibodies (0.5 *μ*g/100 *μ*l) for 20 min in the dark. Thereafter, the slides were washed twice with D-PBS, fixed with 4% PFA for 10 min, and rinsed twice with D-PBS and sterile water. Slides were mounted with Mowiol and allowed to set overnight at room temperature. Cells were visualized under a iCys LSC (Compucyte, Cambridge, MA, 40× objective). Fluorescence of PE and FITC excited with 488 nm laser was measured on iCys LSC apparatus using appropriate set of filters. Control samples stained with FITC (LTA) or PE (ChOD) separately were used for adjustment of measurement parameters. First, the LTA binding regions (green dots) were identified, next two-colour scanning was performed. Spearman correlation coefficient (R2) was calculated for the median and integral intensities of green (LTA) and red (ChoD) fluorescence.
