# Supplementary figures and images for: Cholesterol Oxidase Binds TLR2 and Modulates Functional Responses of Human Macrophages
Source: Mediators Inflamm. 2014 Jul 8;2014:498395. doi: 10.1155/2014/498395 (PMC4121183; doi:10.1155/2014/498395)

**A**

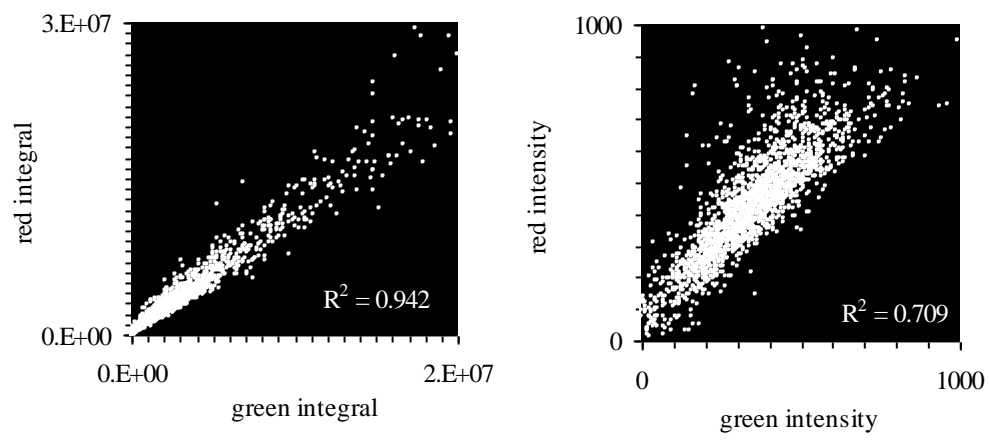

**B**

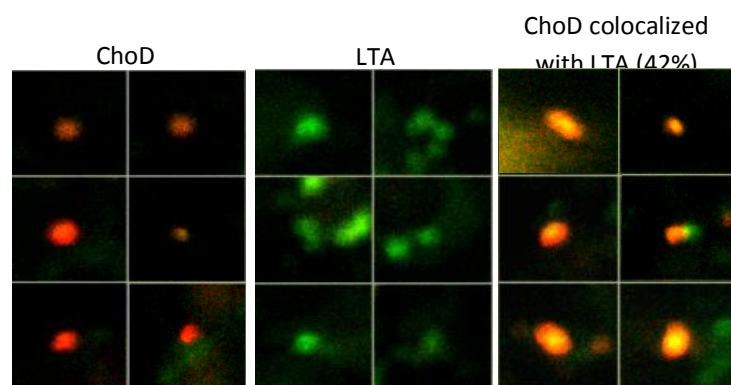

Figure S1

Supplement: Supplementary file 1 — Laser-scanning cytometry (LSC) analysis: THP-1-derived macrophages were incubated briefly (30 min) with ChoD (0.5 U/ml) and LTA (100 μg/ml) at room temperature. After incubation, cells were gently washed three times with 5% FBS in D-PBS and stained with primary antibodies against N. erythropolis ChoD (rabbit polyclonal antibody, 20 μg/100 μl) and LTA (mouse mAb, 1 μg/100 μl) in D-PBS for 30 min at room temperature. Non-specific binding was blocked by incubating with 10% FBS in D-PBS for 20 min. Samples were then gently washed three times with 5% FBS in D-PBS and incubated with FITC-conjugated anti-mouse or PE-conjugated anti-rabbit secondary antibodies (0.5 μg/100 μl) for 20 min in the dark. Thereafter, the slides were washed twice with D-PBS, fixed with 4% PFA for 10 min, and rinsed twice with D-PBS and sterile water. Slides were mounted with Mowiol and allowed to set overnight at room temperature. Cells were visualized under a iCys LSC (Compucyte, Cambridge, MA, 40× objective). Fluorescence of PE and FITC excited with 488 nm laser was measured on iCys LSC apparatus using appropriate set of filters. Control samples stained with FITC (LTA) or PE (ChOD) separately were used for adjustment of measurement parameters. First, the LTA binding regions (green dots) were identified, next two-colour scanning was performed. Spearman correlation coefficient (R2) was calculated for the median and integral intensities of green (LTA) and red (ChoD) fluorescence. [file 498395.f1.pdf]
